# Allergen labelling: Current practice and improvement from a communication perspective

**DOI:** 10.1111/cea.13830

**Published:** 2021-01-26

**Authors:** W. Marty Blom, Liselotte M. van Dijk, Anouska Michelsen‐Huisman, Geert F. Houben, André C. Knulst, Yvette F. M. Linders, Kitty C. M. Verhoeckx, Bregje C. Holleman, Leo R. Lentz

**Affiliations:** ^1^ TNO The Netherlands Organisation of Applied Scientific Research Utrecht The Netherlands; ^2^ Department of Dermatology and Allergology University Medical Center Utrecht Utrecht University Utrecht The Netherlands; ^3^ Utrecht Institute for Linguistics OTS Utrecht University Utrecht The Netherlands; ^4^ Center for Translational Immunology University Medical Center Utrecht Utrecht University Utrecht The Netherlands; ^5^ Present address: Nivel Netherlands Institute for Health Services Research Utrecht The Netherlands; ^6^ Present address: Department of Dietetics St Jansdal Hospital Harderwijk The Netherlands

**Keywords:** anaphylaxis, food allergy, prevention, quality‐of‐life, regulatory aspects

## Abstract

**Background:**

Allergen information on product labels is crucial in food allergy management, though inadequacy in current labelling practices is one of the major causes for accidental reactions upon consuming prepacked food products.

**Objective:**

This study analyses current status of communicating allergen information on food labels and provides practical recommendations for improving the label format based on communication theory.

**Methods:**

Product labels (*N* 288) of seven food categories from private label products and brands were obtained at three retailers in the Netherlands. Information regarding the 14 EU‐regulated allergens was evaluated by the frequency of emphasizing allergens in the ingredient list, use of precautionary allergen labelling (PAL), icons and an allergen information section. Effectiveness of communication was assessed evaluating readability and findability of information on allergens using principles of Gestalt and Cognitive Load theories.

**Results:**

As requested by EU regulation 1169/2011, emphasizing allergens in the ingredient list was almost 100%, all other presentations of information on allergens on labels was highly diverse. A separate allergen information section was present on most private label products. This section could, but not necessarily did, repeat allergens from the ingredient list and/or give a PAL. Brands often provided a PAL at the end of the ingredient list. Part of the products displayed an icon at different locations of the label. Label background, a lack of cohesion and variation in location of topics hamper the identification of relevant information on allergens by (allergic) consumers. Recommendations include a standardized order for mandatory and voluntary topics on the label and a separate allergen information section.

**Conclusion and clinical relevance:**

Overall, consumers encounter a wide and inconsistent range in ways of presentation of allergen information on labels. Standardization according to basic design principles can improve usability and support safe food purchases for allergic consumers.

## 1 INTRODUCTION

Food allergy is a worldwide health problem with regional differences in the reported prevalence from 0.3 up to 10%.^1^, ^2^ Though prevention of developing a food allergy and development of therapies to cure food allergy are promising, the best option for allergic consumers still is to avoid ingestion of the specific food.^2^

Regulation in many regions requires the labelling of allergenic food ingredients when used in food manufacturing. In addition to this, cross‐contact with allergens can occur at many stages of the food production chain. Despite all management measures taken, food manufacturers sometimes cannot guarantee the absence of unintended allergens in the final product and may apply a Precautionary Allergen Labelling (PAL).

Unexpected allergic reactions frequently occur and mostly range from mild to severe.^3^ Several factors have been mentioned to contribute to the risk of unexpected reactions. Some of these are related to food manufacturing (undeclared ingredients, wrong label on pack, lack of a PAL statement or incomplete PAL statements). Other factors are consumer‐related (ignoring or missing PAL, not reading or failure to read labels or missing the allergen as an ingredient). However, the contribution of each factor to the risk of unexpected reactions is not exactly clear.^4^, ^5^, ^6^ For food‐allergic consumers, the label on food products, and especially the allergen information part, is an important and often crucial communication tool to avoid allergen(s). Regulation (eg EU No 1169/2011) prescribes the declaration of allergenic ingredients if used in food production. However, it gives only limited directions on how this should be presented on the pack, which leaves room for flexibility in use by individual food business operators. The use of PAL is voluntary and consequently flexibility in use and presentation form applies. Similarly, providing an allergen information box or using icons are not clearly defined in EU Regulation.^7^ Further, country‐specific interpretations and guidelines exist.^8^, ^9^, ^10^ In a global market, an allergic consumer might thus be confronted with different presentation forms of information on allergens.

As with all mandatory communication, there can be a gap between official guidelines and everyday practice.^11^ Besides informing customers on the mandatory topics for food safety and quality, food business operators use the label to persuade customers to buy their products. Research shows that allergic consumers experience difficulties to identify, locate, read and understand the allergen information on food labels, but further study is needed on how allergy information might be best provided.^12^, ^13^ Studies systematically analysing allergen information on food labels mainly focussed on compliance with legal guidelines, signalling methods of marking allergens on the pack, or the variation of PAL statements used on products.^14^, ^15^, ^16^, ^17^ However, steps to improve the functionality of communicating allergen information on food labels for food‐allergic consumers and those buying for an allergic consumer have to our knowledge never been investigated.

In the present study, the communication about allergens on food labels is investigated with emphasis on the functionality for (allergic) consumers and on identification of the specific topics for improvement in allergen labelling design. The study does not assess the compliance of allergen information on labels to EU or national regulation; instead, it takes a consumer perspective. First, the allergen information currently provided to (allergic) consumers in The Netherlands is quantitatively analysed for a collection of food labels. Second, a qualitative examination using principles of communication graphic design theory, that is, the basic design principles of Gestalt Theory^18^ and Cognitive Load Theory,^19^ are used to systematically evaluate the readability and findability of allergen information. The aim is to provide a set of recommendations for providing allergen information on food products to improve labelling.

## 2 MATERIAL AND METHODS

### 2.1 Data collection

Packaged food products were collected for evaluation of (precautionary) allergen labelling regarding the 14 EU‐regulated allergens.^7^ Six graduate students of Utrecht University were recruited to collect the labels. The collection was performed in May and June 2018. The students were instructed to take photographs of the label with specific emphasis on the ingredient list and the possible additional separate allergen information, either a PAL statement, an icon and/or an allergen information section. They registered the product name, category and manufacturer type (either retail or brand). Digital photographs were taken of at least nine products per food category (Table 1) and manufacturer type. Labels from private label products from three major retailers were collected for the selected seven food categories. Together, these retailers are covering 60% of the food store market in the Netherlands. Similar types of products were collected from brands for each of the food categories. Photographs were made at three food stores in the city of Utrecht, and at one store, labels from individual brands were collected.

**TABLE 1:** Number of collected food labels per food category and manufacturer

Food category	Total	Retailer 1 (*n* = 71)	Retailer 2 (*n* = 71)	Retailer 3 (*n* = 70)	Brands (*n* = 89)
Bread toppings	42	10	10	10	12
Fast foods	40	10	10	10	10
Breakfast products	41	10	10	10	11
Snacks	56	10	10	10	26
Soup products	39	10	10	10	9
Desserts	42	10	10	10	12
Dinner meal products	41	11	11	10	9

### 2.2 Food categories

Seven categories were defined with food products that are consumed on a regular basis and at specific eating moments during a day. These categories were as follows: breakfast products such as cereals, rusk, flakes; products typically eaten as a snack (including crisps, nuts, chocolate bars and cookies); fast foods (including pizza, fried shrimp, quiche, sausage roll and cheese soufflé); soup products (including soup, soup powders and soup vegetables); desserts (yoghurts, custards, ice creams and small cakes); dinner meal products (frying oil, wraps, rice, sauces and meal boxes); and bread toppings (including cheese, sweet sprinkles, peanut butter, salads and meat products). If possible, similar types of products were selected for the private label products of the different retailers and the brands. Table 1 presents the distribution over these categories of 301 collected food labels.

### 2.3 Quantitative analysis

Data were entered into an Excel database accompanied with a link to the photograph of each product. Photographs were analysed for the list of ingredients, the PAL, icons, and the presence of a separate allergen information section. The ingredient list was examined for the presence of allergens and the various ways of emphasizing these allergens. Information on the possible PAL for the phrasing and location was included in the database. Three types of expressions were observed with only minor variation within each of the types and therefore classified into *May contain X*, *May contain traces of X*, or *Produced in a factory*/*production line at which X is also used (shared equipment)*, which is further referred to as a ‘*factory*/*production*’ statement. The use of icons was registered. The presence of a separate allergen information section, its content, comparison with allergens in the ingredient list, and location, for example whether the section was in close proximity to the ingredient list, was collected. This separate allergen information section is defined by the presence of a visual clue by a header (‘allergy information:’), a black line, a clear space or a combination. The final data set was checked independently by three of the authors of this paper. The data set was quantitatively analysed for the current status of allergen information by using descriptive statistics.

### 2.4 Qualitative evaluation

A qualitative analysis was performed on the current allergen labelling practice in order to evaluate the functional communication of food labels for (allergic) consumers. Several of the basic design principles of Cognitive Load Theory^19^ and Gestalt Theory^18^ were applied (Table 2) to describe the communication and to formulate the recommendations for improvement.

**TABLE 2:** The design principles of Cognitive Load Theory^19^ and Gestalt Theory^18^ for evaluation of food labels

**Cognitive Load Theory** ^19^
This theory was developed in the domain of instructional psychology in order to analyse the factors that complicate learning a new task and to develop guidelines for designers of instructional material in order to optimize performance.
***Principles used to analyse the food labels***
The *intrinsic* cognitive load is the level of inherent complexity of the task: learning to cook an egg is easier than learning to prepare lasagna. The *extraneous* cognitive load refers to the often unnecessary complexity of the instructional material. Finding out whether there are any allergens in the boiled egg is easier than for lasagna: assessing the safety of food may create extraneous load when buyers have problems locating allergen information and need to find out whether the lasagna does not contain an allergen. *Germane* cognitive load refers to cognitive activity of the learner that is used to develop schemes in order to process information on a higher level and thus make free capacity in working memory, which is limited in both capacity and duration. A central notion in the Cognitive Load Theory is the differentiation between three types of cognitive load. When all food labels would have the same order of information, consumers would gradually construct a scheme of relevant food topics, which means only a temporary increase of germane load. At the same time, long‐term extraneous load would decrease, because locating allergen information would become the easiest part of reading food labels.
**The Gestalt Theory** ^18^
This theory was developed in the early 20th century by three German psychologists (Wertheimer, Koffka and Kohler) who tried to formulate some basic principles of interpreting visual stimuli. These principles played an influential role in thinking about graphic design,^32^ and help to organize the visual field of the food label in ways that support the main goals of food product information
***Principles used to analyse the food labels***
**Figure‐Ground prinicple**: people perceive a visual element always as being in the foreground or part of the background. Foregrounded elements are interpreted as being prominent (the figure) if other elements recede into the back (the ground). The principle of **Grouping**, also presented as Common Region: when objects are located within the same region of the food label, consumers will perceive these elements as belonging semantically together. These elements may share common aspects or purpose. The principle of **Proximity**, which is related to the second principle: elements presented closely together appear to be more related than elements that are spaced apart. So, within one group the order of the topics is meaningful. This principle helps to think about the structure of the different topics presented on the food label.

## 3 RESULTS

### 3.1 Data set

In total, 301 unique labels of manufactured food products were collected. In 288 of the labels, information relevant for the allergic consumer was present, for example an ingredient list, an allergen information section, a PAL or an icon. Thirteen labels did not contain any information on any of the 14 EU‐regulated allergens and were excluded from further analysis.

### 3.2 Quantitative analysis of allergen information

#### 3.2.1 Emphasizing allergens in the ingredient list

There was a significant preference (80%) for emphasizing the allergenic ingredients using bold‐face type (Table 3). Capital font was used significantly less. Among the brands, more variation was observed, including the use of a combination of bold and capital font type and underlining. Retailers did not use underlining. There was one case where a product contained an allergenic ingredient without any highlighting. Further, retailer 1 occasionally highlighted allergenic ingredients in blue and bold to stand out to the other ingredients in black letter type. Italic font of allergenic ingredients was never used.

**TABLE 3:** Types of marking allergens in ingredient lists per manufacturer (*n* = 276)^a^

Marking	Total (%)	Number of products *n* (% per manufacturer)			
		Retailer 1 *n* = 65	Retailer 2 *n* = 64	Retailer 3 *n* = 69	Brands *n* = 78
Bold	222 (80.4%)	63 (96.9%)	51 (79.7%)	51 (73.9%)	57 (73.1%)
Capitals	40 (14.5%)	1 (1.5%)	13 (20.3%)	18 (26.1%)	8 (10.3%)
Bold + Capitals	11 (4%)	0 (0%)	0 (0%)	0 (0%)	11 (14.1%)
Underlining	2 (<1%)	0 (0%)	0 (0%)	0 (0%)	2 (2.6%)
No marking	1 (<1%)	1 (1.5%)	0 (0%)	0 (0%)	0 (0%)

^a^ Eight labels had an ingredient list without any allergens, but either a precautionary allergen statement or an allergen icon. Four labels were from single ingredient products without other ingredients in an ingredient list, but with a separate allergen information section. These twelve labels were excluded from the set of 288 labels for this part of the analysis.

#### 3.2.2 Use of PAL statements

Half of the collected labels (52.1%) contained a PAL statement to warn for a possible unintended presence of allergens, and these were placed either following the last ingredient in the ingredient list or in a separate allergen information section. For those labels with a PAL, the ‘may contain traces’‐ statement was used most frequently (76% overall score). Retailers 2 and 3 only used this statement, whereas retailer 1 also used the ‘factory/production’ statement on 48% of its labels with a PAL. Both statements were used within the same product categories. In contrast to this observation, within the brands, all three different PAL statements were found, 58% was a ‘may contain traces of X’ statement followed by ‘may contain X’ (36%) and the ‘factory/production’ statement in just 6%. One product label even contained two different formulations of a PAL statement: ‘This product may contain peanut and traces of egg, gluten and nuts’.

#### 3.2.3 Presence of allergen icons

The icons present on labels mentioned whether a product was ‘free from’ milk or gluten, that is none of the icons were used to indicate the (possible) presence of milk or gluten. Icons for other allergens were not used at all. Icons were present on 21.9% of all 288 labels. This was due to private label products of retailers 1 and 3 that displayed an icon on 49.3% and 38.6%, respectively, of their product labels. Retailer 2 and almost all brands were consistent in not using icons for allergens. Only one brand label displayed a gluten‐free icon.

#### 3.2.4 Presence of a separate allergen information section

In 71% of all labels (204 out of 288), a separate allergen information section was present. This means that this section was visually separated with a line, open space or another visual marking, like a bold heading *Allergen information*. Most private label products of retailers displayed such a distinct section, whereas on 22.8% of the brand labels, a separate allergen information section was present.

#### 3.2.5 Content of the allergen information section

Most, but not all, allergen information sections repeated the allergenic ingredients from the ingredient list (Table 4). This means in case such labels displayed only a PAL in the allergen information section, two situations: either allergens were not present as an ingredient (products of retailers 1 and 2) or allergens were present and marked in the ingredient lists (products of retailer 3 and brands).

**TABLE 4:** Presence and content of the allergen information section

	All labels (*n* = 288)	Retailer 1 (*n* = 71)	Retailer 2 (*n* = 68)	Retailer 3 (*n* = 70)	Brands (*n* = 79)
Presence^a^	204 (70.8%)	71 (100%)	64 (94.1%)	51 (72.9%)	18 (22.8%)
Content					
Allergens	96 (47.1%)	42 (59.2%)	38 (59.4%)	14 (27.5%)	2 (11%)
Allergens + PAL	86 (42.2%)	27 (38.0%)	23 (35.9%)	35 (68.6%)	1 (6%)
PAL only	14 (6.9%)	2 (2.8%)	3 (4.7%)	2 (3.9%)	7 (39%)
Reference to ingredient list	1 (0.5%)	0 (0%)	0 (0%)	0 (0%)	1 (6%)
Reference to ingredient list + PAL	4 (2.0%)	0 (0%)	0 (0%)	0 (0%)	4 (22%)
Other	3 (1.5%)	0 (0%)	0 (0%)	0 (0%)	3 (17%)

^a^ Single ingredient products, like oatmeal or cottage cheese, were included, because they can have an allergen information section and/or a PAL.

Allergenic ingredients were repeated in the allergen information section, but they could be named and/or highlighted differently. An example: the ingredient list sometimes mentioned **wheat** flour; **cream, soy**bean, and the allergen information section cited this as wheat gluten, lactose, milk protein and soy. Sometimes, these were emphasized in bold as well.

A few brands used the allergen information section to refer to the ingredient list. An example: ‘For allergens, including cereals containing gluten, see **bold** ingredients’. The category ‘other’ contained a PAL and a reference to a website for more allergen information, and a combination of a list of allergens including a gluten‐free statement.

### 3.3 Qualitative evaluation of the label design

The quantitative results do not give a complete insight into the usefulness of the communication for the (allergic) consumer. In the following sections, the effectiveness of current labelling practices is qualitatively evaluated by discussing the design of allergen labelling information using theoretical communication principles (see Material and Methods).

#### 3.3.1 Readability of food labels

The quantitative analysis revealed that different ways were used to emphasize allergens in the ingredient list. Considering the Gestalt Principle of Figure‐Ground, humans always select some elements in a picture or a graph, as the **figure**, being the object of interest, against a back**ground**, on which the figure rests. A **bold** presentation of the allergenic ingredients in the ingredient list seems to be preferable, because the contrast with the background increases.

**FIGURE 1:**
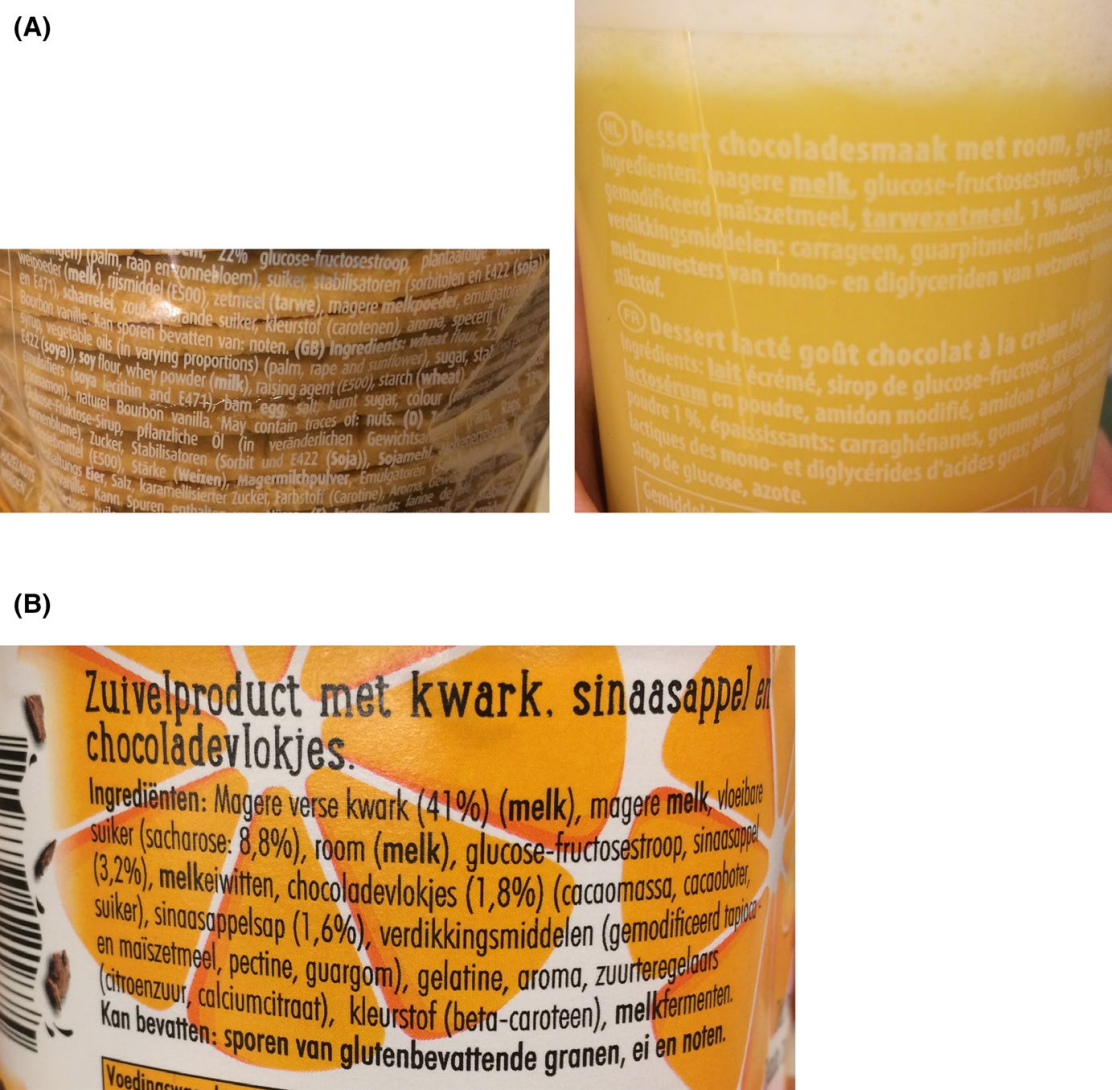
Examples of food labels demonstrating figure‐ground problems consumers will have in finding and reading the allergens due to (A) transparent packaging and (B) creative background design.

In several food labels, the lack of contrast between background and text hampers to distinguish bold ingredients from other ingredients (Figure 1). The two labels in Figure 1A are printed on transparent packaging, causing low contrast between figure and ground. The label in Figure 1B has text printed on a creative background design, causing a cluttered background with different white and orange regions.

**FIGURE 2:**
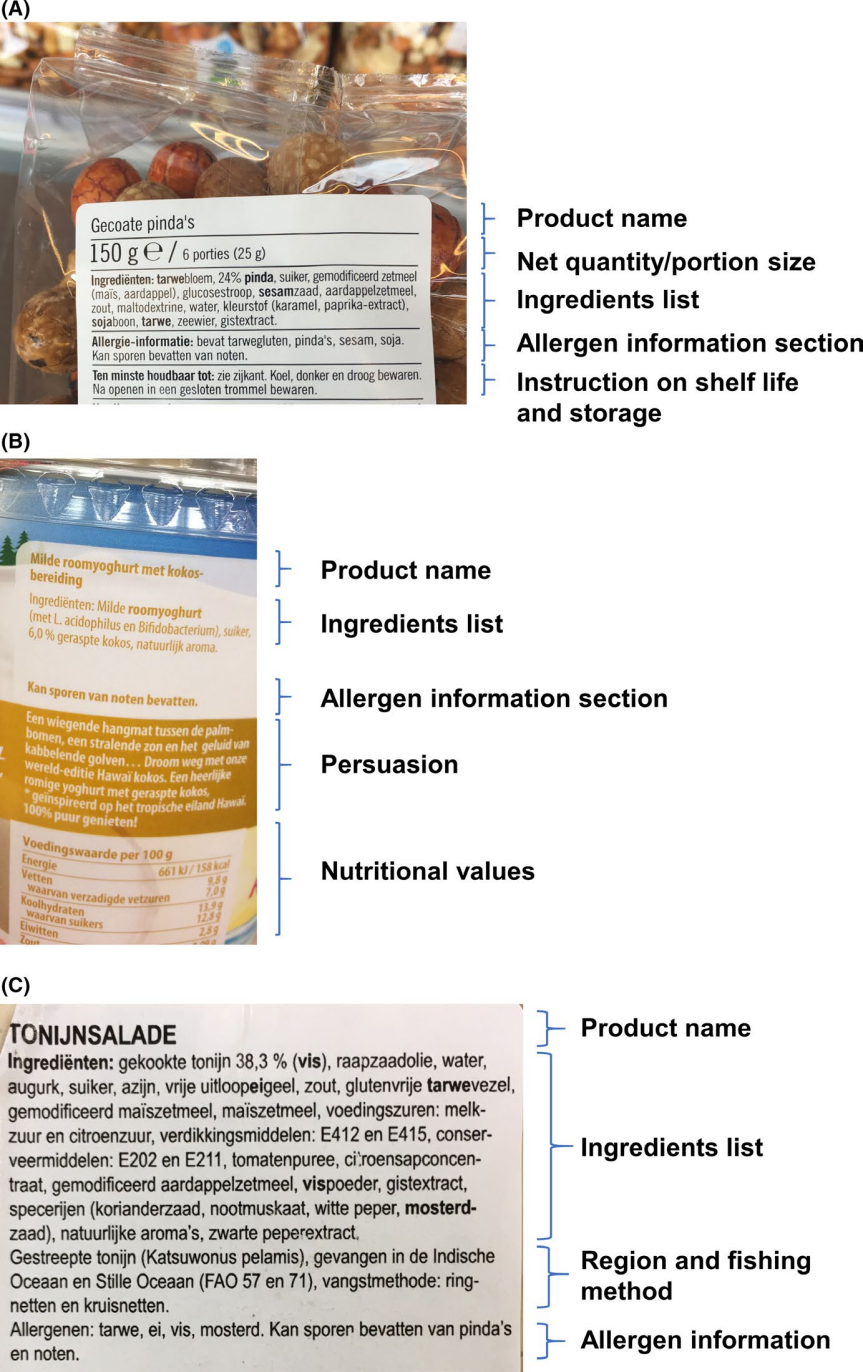
Labels evaluated using theoretical communication theories. A, Transparent plastic package with good Figure‐Ground representation for finding allergenic ingredients in the ingredient list. Information units are visually separated using black lines. The ingredient list and the allergen information section are grouped in a Common Region, which strengthens usability for readers. B, Information units are grouped visually by the white background, and topics on allergens have a common region. In the orange text box, however, an interfering persuasive unit has been inserted, a rather long narrative creating a Hawaiian atmosphere with hammocks, palm trees, the sun and rippling waves, interfering with mandatory related topics. C, Food label for a tuna salad not matching the principles of Proximity and Grouping. The unrelated information on region and method of fishing interferes with the allergen information presented in the ingredient list and allergen information section. Allergenic ingredients are highlighted in bold; however, additional allergen information is not clearly visible.

The decision for packaging in transparent plastics does not automatically need to result in low figure‐ground discrimination of ingredient lists, as can be seen in Figure 2A. A smooth white background is used to discriminate the text from the coated peanuts inside the package, providing sufficient figure‐ground contrast for reading the ingredient list with allergens presented in bold.

#### 3.3.2 Grouping of allergen information

The Gestalt Principle of Grouping or Common Region states that information units that are visually grouped together are interpreted as belonging semantically together. Readers expect some connection between these units. For (food‐allergic) consumers the ingredient list, a separate allergen information section, a PAL statement and an icon, are best understood and located when they are presented in a common region of the visual space of the food label. Figure 2 shows two food labels with a separate allergen information section clearly standing out, and visually connected with the ingredient list because of their proximity to each other. Overall, in 86% of the labels, this allergen information section was in proximity to the location of the ingredient list (Appendix S1 Table A1, Figure A1). But for all food manufacturers, we observed that on some of their product labels the allergen information section was separated or interrupted by other information, such as preparation instructions, text explaining the origin of ingredients or the product, or mentioning sustainability (Figure 2C, Appendix S1 Figure A2).

Most brand labels (77%) did not have a distinct allergen information section (Table 4). These labels marked the allergens in the ingredient list, and if they provided a PAL, it followed directly after the last ingredient (Appendix S1 Figure A3). Although this meets the Grouping principle, without a visual clue, this PAL may be easily overlooked.

Most icons (80%) were displayed in close proximity to the ingredient list, but the location varied: the icon was either above or below the ingredient list and could be in or separate from the allergen information section (Appendix S1 Figure A1). In one case, the icon was on a different side of the package (Figure A1‐G). The images used differed between the producer types but were standardized within a retailer.

#### 3.3.3 Cohesion of different topics

The Gestalt Principle of Proximity suggests that perception is facilitated when related topics such as ingredients, allergen information and nutritional values of these ingredients are grouped together in proximity. Persuasive information sometimes interferes, like in Figure 2B,C. Although the visual design of Figure 2B with different background colours might sufficiently relate the allergen information with the nutritional values, right between these units, a rather long text fragment has been inserted. Relevant food information topics should not be cluttered with persuasion or other unrelated optional topics, because this negatively affects cohesion.^20^

Figure 2C shows another food label that does not match with the principles of Proximity and Grouping. There is a title **TUNA SALAD**, followed by a header **Ingredients** and a text fragment in three different paragraphs. Unlike on the food labels in Figure 2A,B, these groups are not visually separated by horizontal lines or background colours and there is no visual marking for the allergen information section. The principle of proximity is not followed, because an extra topic (the fishing region and method) is presented between ingredient list and the allergen information section. This design creates extraneous cognitive load, because extra energy is needed in locating relevant allergen information.

#### 3.3.4 Consistency in the order of topics

Food labels may be considered as a *genre*, like recipes, patient information leaflets, manuals and weather forecasts. Consumers recognize such documents easily and immediately create a set of genre‐related expectations. In case of a recipe, they expect a fixed structure: a list of ingredients at the start, followed by a set of instructions.^21^ These expectations are part of the scheme, generated by spending germane cognitive load, in order to decrease extraneous load. For food labels, consumers would be helped to create such a scheme when topics that semantically belong together were visually located in close proximity and presented in a fixed order of topics. Consistency in the order of presentation of the mandatory and voluntary topics would help allergic consumers and those buying for an allergic person to locate relevant information most efficiently (see Table 5 for the complete list of all topics—mandatory and optional—found on food labels). In our data sets, retailer 1 creates a relatively high level of consistency for the topic order in the design of their private label products, but also within this group of products, the location of the allergen information is not consistently placed directly below the ingredient list (see examples in Appendix S1 Figure A1).

**TABLE 5:** Topics on food labels from EU Regulation no 1169/2011^a^

Mandatory^a^	Optional/voluntary
name of the product list of ingredients allergen information^b^ net quantity of food in package shelf life (best before) instructions for (storing, eg cooled) instructions for use (eg shake before use) name and country of producer origin nutritional values	PAL^b^ icons^b^ instructions for preparation health information region of production way of production (sustainability) promotion of product

^a^ EU Regulation No 1169/2011 of the European Parliament and of the Council of 25 October 2011 on the provision of food information to consumers for exact legal wording and all requirements.

^b^ Allergen information is mandatory through marking ingredients in the ingredient list, however an allergen information box, an icon and mentioning a PAL is voluntary and need implementing acts in the future (Regulation (EU) 1169/2011 Article 9, Article 21, Article 36). Further country‐specific interpretations might exist.^8^, ^9^

The group of brands consists of many different food manufacturers. Consequently, this group shows a large variety in presentation formats. Although the name of the product is almost always presented on top of the label, all other mandatory and optional topics were found in any order (examples in Appendix S1 Figure A4).

## 4 DISCUSSION AND RECOMMENDATIONS FOR ALLERGEN LABELLING

Based on an analysis of nearly 300 labels, we conclude that allergic consumers and people buying food for allergic patients encounter a wide variety in ways of communication of allergen information on the labels of food products with one exception: almost 100% of all food labels emphasized allergens in the ingredient lists using bold and capital font. The *readability* on transparent or decorated labels could be enhanced by creating more contrast with the background. A PAL statement, an allergen information section and/or a ‘free from’ icon were mostly grouped with the ingredient list in a common region of the label; however, these units were not always in proximity to each other, thereby hampering the identifying of relevant information. In addition, *findability* of information on allergens among all mandatory and optional topics provided on a product label was reduced by a lack of cohesion in the topics and topic order. Overall, the lack of standardization in content, order, and design of allergen information in combination with all other mandatory and optional topics on labels across brands and retailers, reduces the *usability* for consumers in easily locating and interpreting this information.

Our study shows that label designs of brands varied largely between brands and deviated from the label designs of the three private labels. The brand labels are from a highly miscellaneous group of food manufacturers, each with their own labelling policies. In contrast, labels of each of the retailers were a rather homogenous group, very likely because a retailer prescribes one labelling policy for their range of different private label food products. Still, within private labels variation in allergen information is present. A different selection of labels in our study, with more labels of brands, or of other retailers, would probably have altered the proportions found in our study. But this is not expected to change the general findings of our study regarding the variability in what and how allergen information is communicated. The three retailers have 60% of the market store share in the Netherlands, and they represent a considerable part of the food information that is offered to customers in The Netherlands. The study therefore provides a good overview of the range of labels (allergic) consumers encounter when buying food.

Our study is the first to investigate the allergen information provided on labels from a communication perspective. Besides a relatively clear standardization of emphasizing allergens in the ingredient list using a different font type, our study shows that content, location and design of allergen information on the label is very diverse. Previously, allergic consumers have expressed that they experience many difficulties in reading and interpreting food labels, and that standardization of allergen information is very important to them.^12^, ^13^, ^22^, ^23^, ^24^ Still the communication of allergen information present on the examined labels proved to be far from optimal. Based on our evaluation of the current labelling practices using communication theories, we propose six recommendations in order to improve the communication of allergen information.

**FIGURE 3:**
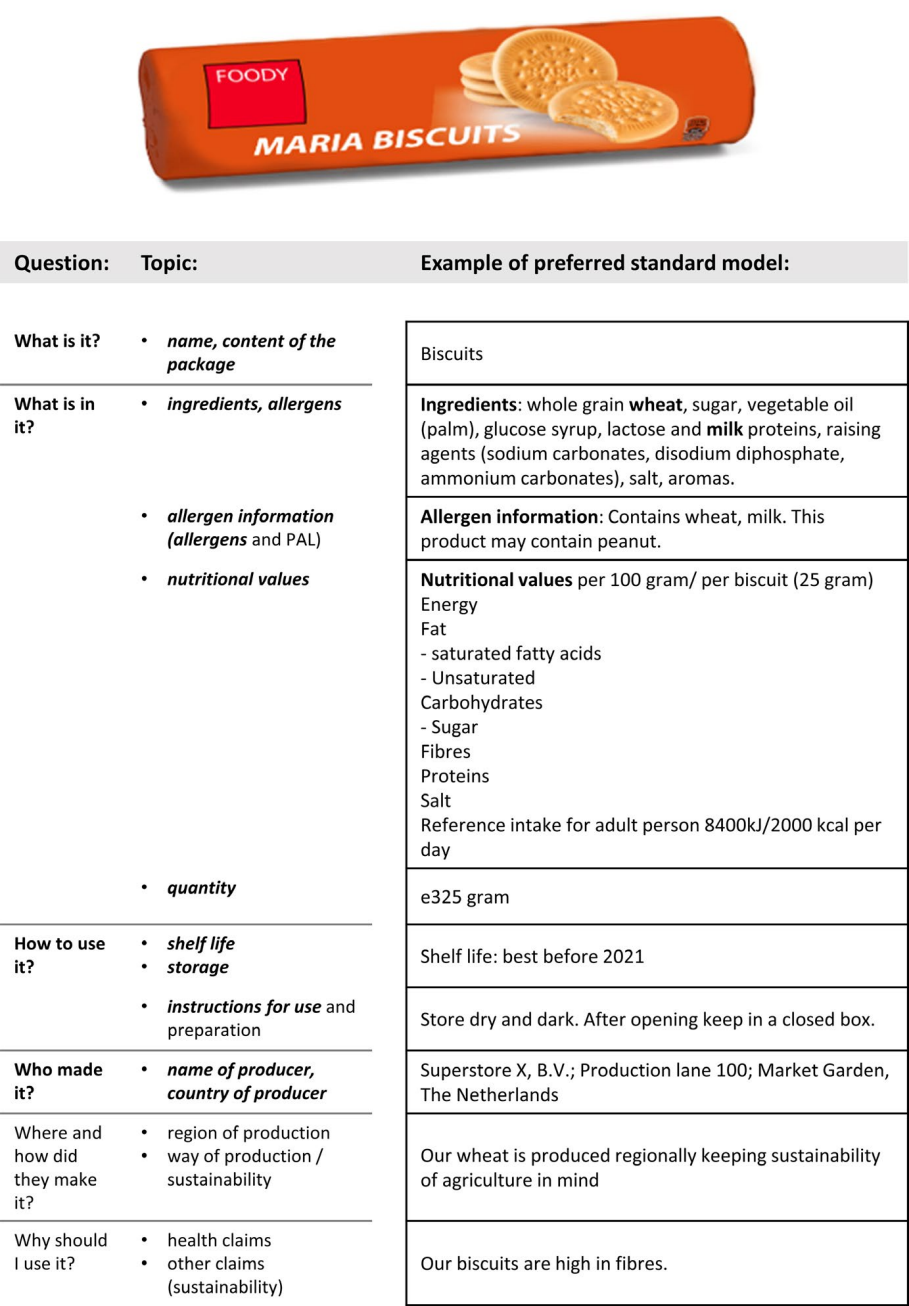
A proposal for a uniform topic order, structured by questions. Mandatory topics are in bold and italic

1. *Ensure all food information is readable (good figure*‐*ground)*. For good readability, visual clutter on background should be avoided and in case of transparent packaging, a white background is preferred for optimal visibility/discrimination.
2. *Presenting allergens in the ingredient list in* ***bold*** *is highly preferred*. In information design, bold is seen as more appropriate than italics for headings by making words stand out.^25^ Also in the design of nutrition labels, the bold presentation of nutrition information and presentation on a white background were shown to be important to improve the uptake of the information by consumers.^26^
3. *Provide grouping of related topics and a uniform topic order on the label*. A basic communication principle in label design is that related topics should be grouped in order to improve information retrieval.^18^ This can be obtained if all topics on allergens are connected in the design of the food label. Additionally, a uniform topic order is preferred. Figure 3 presents an example of a topic order for a label that could be preferred from a communication perspective. This example presents all mandatory topics as well as closely related additional allergen information currently not implemented in EU 1169/2011 regulation yet (PAL, icons, allergen information section). These related topics should not be interrupted or separated by any optional information on the product. Our study suggests that labels may be improved from an allergen information communication perspective by restructuring the order as proposed in Figure 3. It would be interesting to conduct user tests among (allergic) consumers for this proposed format.
4. *Provide an allergen information section*. One way of ensuring cohesion in communication is presenting all allergen related information, for example including the allergens present as ingredient and the PAL (and possible icons), in a separate section after the ingredient list. However, at the moment the voluntary repetition of allergens outside the list of ingredients, either by using the word ‘contains’ followed by the name of the substance or products or by using symbols or text boxes is not allowed in Europe,^27^ though can be in other regions.^28^ From a communication perspective, a separate allergen information section that is clearly standing out from the total label information has advantages for (allergic) consumers in quickly finding the relevant information. Allergic consumers indeed express a preference for a separate allergen information section.^13^, ^22^
5. *Use of one statement for PAL*. Different PAL statements are currently used on food products and may be interpreted as indicative of differences in risks posed by these products. This perception may occur among patients, healthcare professionals^15^, ^29^ and food manufacturers (Linders et al, in prep). Statements related to ‘factory/production line’ or ‘traces of’ are ambiguous in their meaning, leaving room for interpretation (see also Holleman et al. in prep) and should therefore be avoided.^12^, ^13^ A most simple uniform wording like ‘May contain X…‘^9^ or ‘May be present: X…’,^30^ harmonized across all food manufacturers, retailers, product groups and countries, is therefore preferred to avoid individual and more importantly incorrect risk interpretation (Holleman et al, in prep).
6. *Use of allergen icons*. Consumers indicate a preference for icons, sometimes in combination with written text.^12^, ^13^, ^22^ In our set of labels, icons were solely used to indicate the product is gluten‐free or free from milk. At present, icons are not allowed according to EU regulation 1169/2011.^27^ From a communication point of view, development of standardized icons can have advantages when producing food labels for a global food product market. Currently, the same information is repeated in multiple languages for products marketed in various countries, which is highlighted as a source of irritation by consumers.^22^ The use of pictograms will reduce this repetition and can have the advantage of a more efficient use of valuable space on the label. Several local initiatives to develop allergen icons have been published (https://www.foodprotection.org/resources/food‐allergen‐icons/ or by https://www.allergenenconsultancy.nl/producten/iconen‐en‐stickers). However, development of specific icons for allergenic foods needs further investigation to ensure a global common understanding to avoid the risk for an allergic reaction^31^ and avoid confusion with the way free from icons are currently used.

### 4.1 Final remarks

Several of the above recommendations can be applied relatively easily by individual food business operators to improve their communication (provide a good figure‐ground, group all allergen information in proximity and do not place unrelated other information in between). For other recommendations, like the standardized topic order, the use of universal icons, or the use of a separate allergen information section, and the preferred PAL statement, further studies should help to determine the optimal content and design. The preferences and needs of all stakeholders involved, including (allergic) consumers, food business operators and regulators, should be taken into account, and would need regulatory acceptance and harmonization to implement.

Overall, we conclude that standardization and application of basic design principles would considerably improve usability of allergen information and would support safe food purchases for allergic consumers.

## CONFLICT OF INTEREST

The authors declare no conflict of interest.

## AUTHOR CONTRIBUTIONS

BH, LL, GH, AK and MB contributed to the conception and design of this study. LD and AM substantially contributed to the data curation and analysis. MB and LL provided the interpretation of the data and drafted the article. MB, LL, BH, GH, AK, KV and YL contributed in critically interpret and revising the article. All authors have read and approved the final manuscript.

## Supporting information

Supplementary Material

## Data Availability

The labels analysed during the current study are not publicly available due to copyright, but are available from the corresponding author on reasonable request.
