# Supplementary material for: Allergen labelling: Current practice and improvement from a communication perspective
Source: Clin Exp Allergy. 2021 Jan 26;51(4):574–84. doi: 10.1111/cea.13830 (PMC8048984; doi:10.1111/cea.13830)
Supplement: Supplementary file 1 — Supplementary Material [file CEA-51-574-s001.docx]

**Appendix A Supplementary information on labels**

***Allergen information section***

Quantitative analysis of the presence and connection of the allergen information section with the ingredients list on 288 labels of retailers and brands showed that 86% of the sections were connected with the ingredients list (Table A1).

**Table A1 Presence and connection of a separate allergen information section on the label**

|  | **Labels**  **(n)** | **Allergen information**  **section ^a)^** | **Section connected with ingredients list ^a)^** |
| --- | --- | --- | --- |
| **All labels** | 288 | 204 (70.8%) | 175 (85.8%) |
| **Retailer 1** | 71 | 71 (100%) | 65 (91.5%) |
| **Retailer 2** | 68 | 64 (94.1%) | 49 (76.6%) |
| **Retailer 3** | 70 | 51 (72.9%) | 44 (86.3%) |
| **Brands** | 79 | 18 (22.8%) | 17 (94.4%) |

***Grouping and cohesion in presentation of allergen information***

The presentation of information relevant for allergic consumers, such as ingredients, allergen information sections, PALs and icons on labels varies in location and grouping. Allergen sections (black box) are usually right under the ingredients list (Figure A1 -A, B, E, F, G), though can be occasionally at a different location of the label (Figure A1-C, D). Icons (arrow) can be in the allergen information section (Figure A1 - A, C), close to other allergen information ( Figure A1-F), or not in close proximity or irrelevant section (Figure A1 - D, E, G).

**Figure A1 Presentation of grouping and cohesion in allergen information**

**
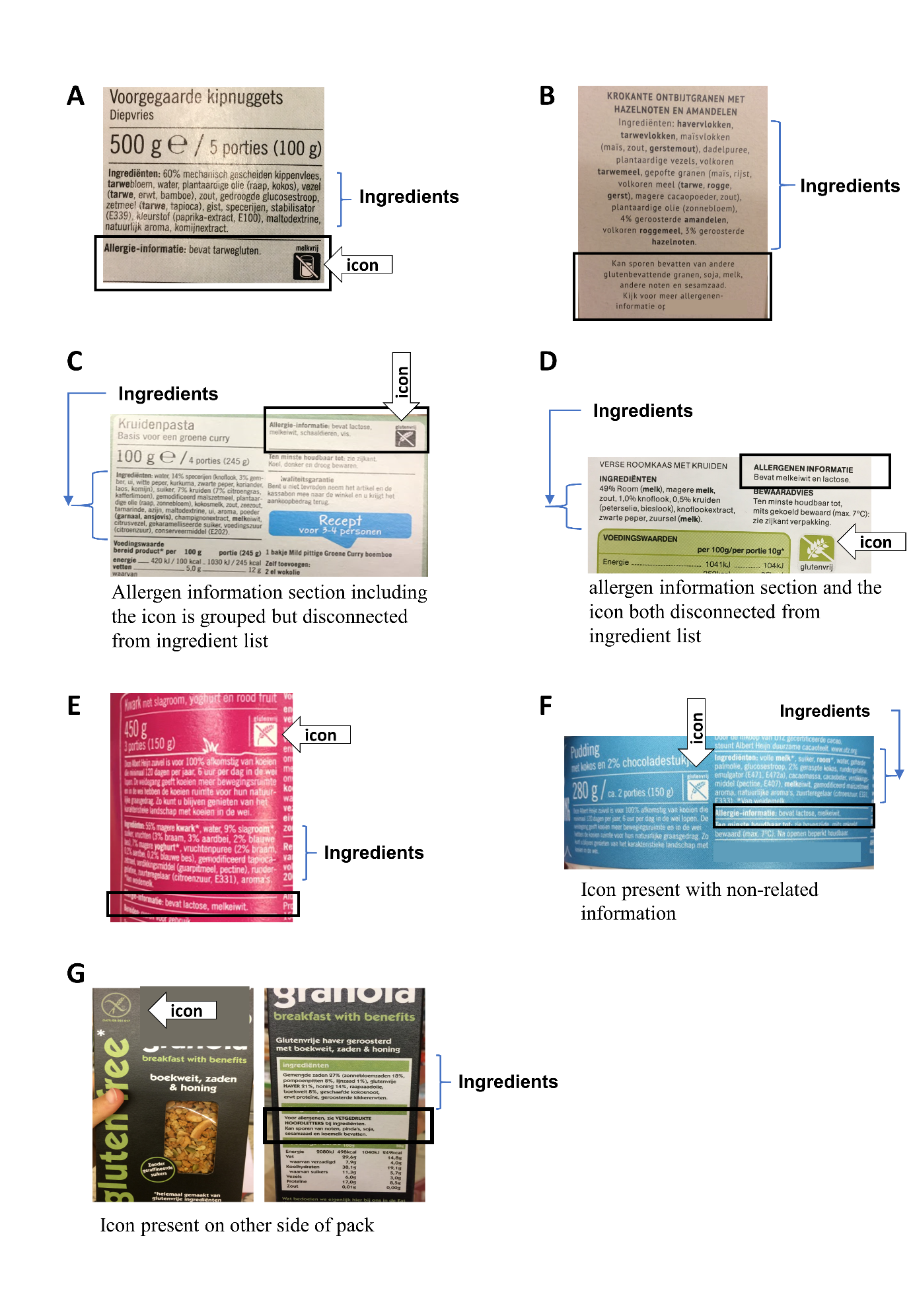
**

**Figure A2 Examples of placing optional irrelevant information between the ingredients list and the allergen information section.**

In case the allergen information section was separated by other information, we found examples of preparation instructions or complaint instructions, text explaining the origin of ingredients or the product as a whole, mentioning sustainability or being fair-trade, or the product being packed under protective atmosphere. A few examples are displayed below.

**
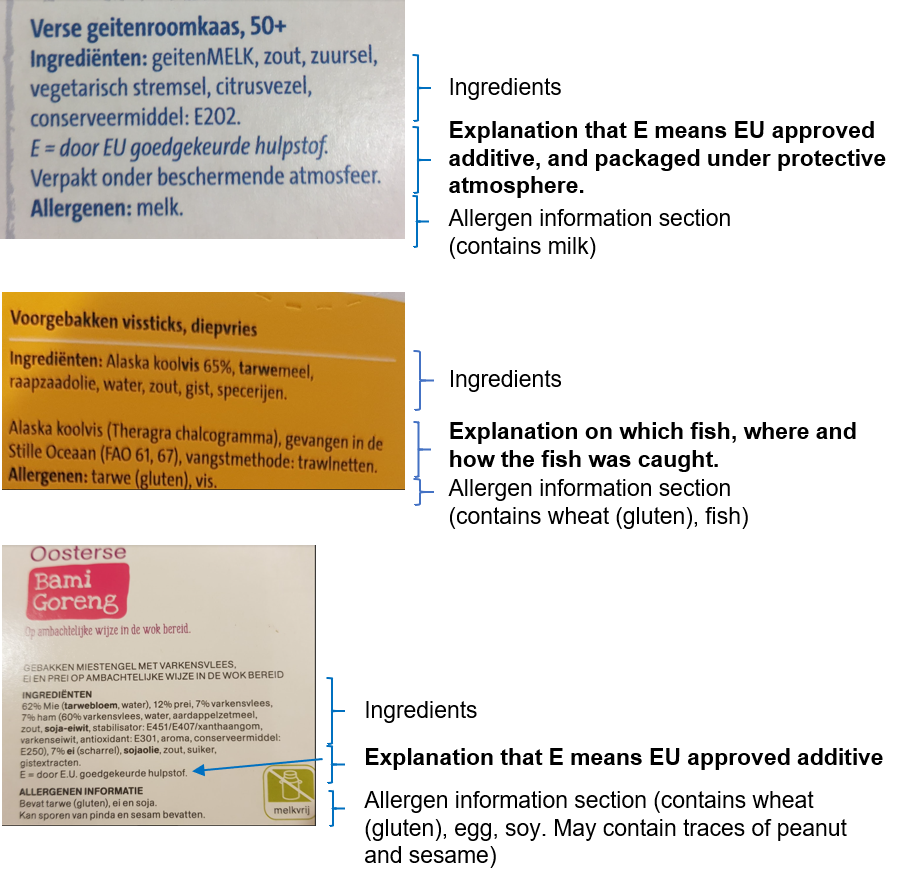
**

**Figure A3 Examples of placing the PAL statement directly after the ingredients list**

Examples showing that PAL statements can be difficult to find. Some labels provide the PAL in bold to support tracing the PAL.

**
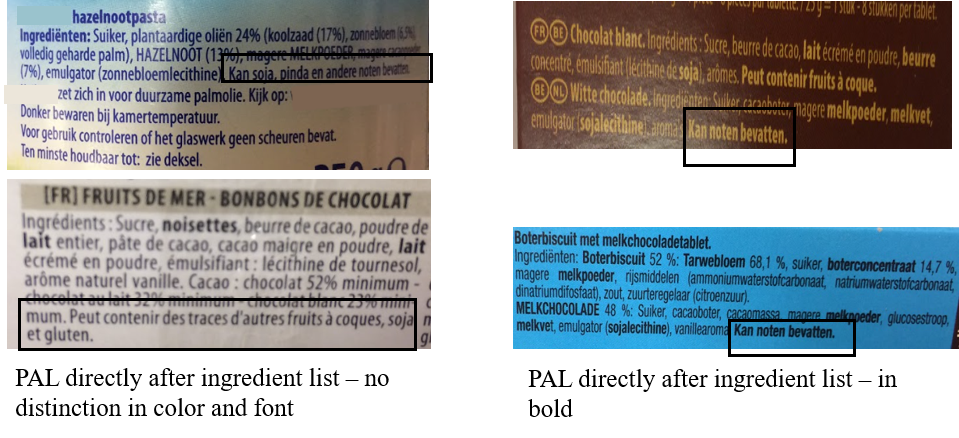
**

**Figure A4 Examples of variance in topic order and information provided among the category desserts of the group Brands**

1 = Ingredients

2 = Allergen information

3 = Nutritional information

4 = irrelevant information (persuasive information and instructions for use).

**
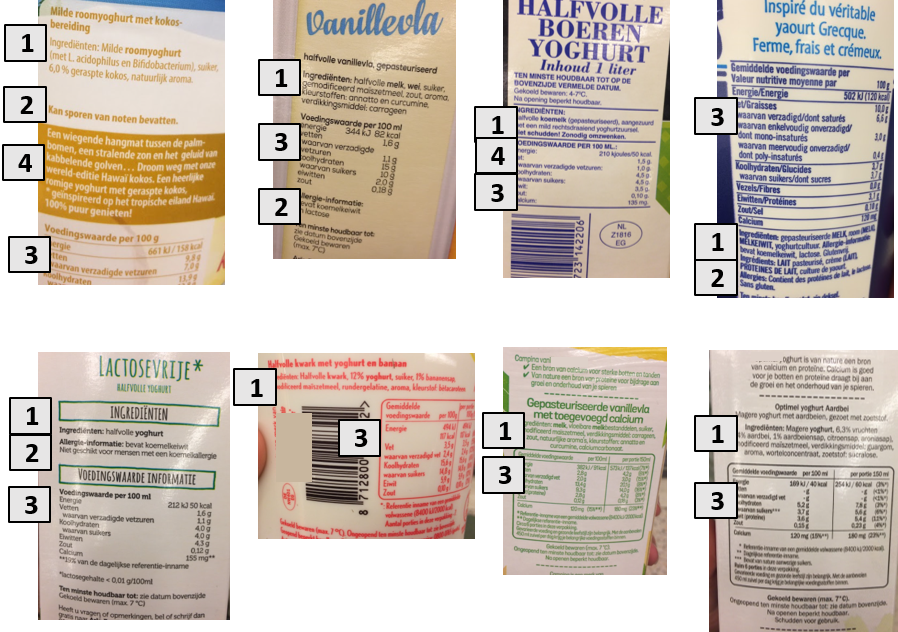
**
